# The relationship of Careers with People Interactions and Volunteering Between Gender-racial Groups in Late-life

**DOI:** 10.1093/geroni/igab046.2593

**Published:** 2021-12-17

**Authors:** Patrick Ho Lam Lai

**Affiliations:** Boston College School of Social Work, Chestnut Hill, Massachusetts, United States

## Abstract

Prior research showed that ethnic minorities in late-life tended to participate less in volunteering, compared to Whites. Older women, in general, spent more time volunteering than older men in the United States. Previous studies showed that occupational statuses, but have not yet discussed occupation categories, affected older adults' volunteering. The Current Population Survey dataset was utilized in this study to explore the relationship between careers with or without people interactions and volunteering of Americans aged 50 to 85 in an intersectionality lens. Regarding races, older African Americans who worked in occupations requiring human interactions, had almost double volunteering rate than those occupations not requiring these interactions. In respect of genders, compared to older men who worked in jobs requiring human interactions, the volunteer rate of those not requiring human interactions was 81% less. Either older African Americans or older men had more associations between their human interactions in career and their volunteering rate, than other racial groups or gender groups individually. Considering races and genders together, comparing to older Asian men who worked in fields needed interactions with others, the volunteering rate of those who did not work in these fields was 52% less. The association of older Asian men between fields requiring human interactions and volunteering rate was the least, among various gender-racial subgroups. Older adults with different racial-gender identities may face varying experiences in different types of occupations. Social and cultural factors among these identities are discussed to better understand the relationships between careers and volunteering in late-life.

